# Chemical defense mechanisms of soybean genotypes against lepidopterans

**DOI:** 10.3389/fpls.2026.1744490

**Published:** 2026-01-29

**Authors:** Josicleia Oliveira Costa, Victoria P. Peña Arroyo, Yeison Núñez-de la Rosa, Vladimir A. Ballesteros-Ballesteros, Jorge Luis Nisperuza Toledo, Willian Garcia Birolli

**Affiliations:** 1 Institute of Chemistry of São Carlos, University of São Paulo (USP), São Carlos, Brazil; 2 Center for Exact Sciences and Technology, Department of Chemistry, Federal University of São Carlos (UFSCar), São Carlos, Brazil; 3 Faculty of Engineering and Basic Sciences, Fundación Universitaria Los Libertadores, Bogotá, Colombia

**Keywords:** integrated pest management, lepidoptera, natural resistance, secondary metabolites, soy, sustainable agriculture

## Abstract

Soybean (*Glycine max* [L.] Merrill) is one of the world’s most important agricultural crops, playing a strategic role in global protein and lipid production. However, its productivity is severely constrained by defoliating lepidopterans such *as Anticarsia gemmatalis, Chrysodeixis includens, Helicoverpa armigera*, and species of the genus *Spodoptera*, which cause substantial yield losses due to their intense herbivory and remarkable adaptive capacity. Conventional management strategies relying on chemical insecticides provide only partial control and are associated with negative environmental and ecological impacts. Although transgenic *Bt* soybeans have demonstrated efficacy against certain pest species, they exhibit limited toxicity toward *Spodoptera* spp. In this context, the exploitation of soybean genotypes with natural resistance represents a promising alternative within the framework of Integrated Pest Management. This review summarizes the principal chemical defense mechanisms underlying soybean resistance to lepidopterans, emphasizing the role of secondary metabolites, such as flavonoids, phenolics, tannins, and volatile organic compounds, that function as toxic, antinutritional, or repellent agents. Several genotypes, including IAC 100, PI 227687, and PI 227682, have displayed resistance to multiple caterpillar species, establishing themselves as valuable genetic resources for breeding programs. Furthermore, recent studies indicate that environmental conditions, plant developmental stage, and multitrophic interactions strongly modulate the expression of these defense traits. A comprehensive understanding of the chemical interactions within the soybean-lepidopteran system is therefore crucial for the development of more tolerant and sustainable cultivars, reducing dependency on insecticides and slowing the evolution of insect resistance. Future perspectives emphasize the integration of omics technologies, bioinformatics, and biotechnology to elucidate key metabolic pathways and accelerate the generation of resistant soybean varieties, ultimately promoting higher productivity and agricultural sustainability.

## 1 Introduction

Plants and insects have coexisted for more than 350 million years. Throughout this long history of interaction and coevolution, plants have evolved a wide array of defense mechanisms to counteract insect herbivory (War et al., 2018). These mechanisms rely on the recognition of molecular patterns associated with tissue damage, which trigger complex defensive responses (Tanaka and Heil, 2021).

In the case of chewing insects, plants perceive attacks through the specific recognition of chemicals present in the insects’ oral secretions, feces, and oviposition fluids (War et al., 2012; Fürstenberg-Hägg et al., 2013). Such signals activate intricate signaling pathways that lead to the production of defensive compounds, including secondary metabolites and proteins, which exert toxic, repellent, or antinutritional effects on the herbivores (Chen, 2008; Peruca et al., 2018; Wari et al., 2022).

Although plants possess a wide array of defense mechanisms, many insect herbivores have evolved sophisticated strategies to overcome or suppress these defenses, enabling them to exploit different plant tissues (War et al., 2018). The effectiveness of these strategies can be further enhanced by environmental factors, particularly climate change (DeLucia et al., 2012). Recent modeling integrating temperature, insect growth, and metabolic rates predicts that herbivore-driven crop yield losses may increase by 10–25% per °C of global warming in major grain production systems (Deutsch et al., 2018). Consistently, a large-scale global synthesis encompassing 9,682 cases across 1,774 plant species from six continents demonstrated that climate warming is expected to intensify phytophagous insect pressure across extensive regions of the globe (Liu et al., 2024b).

Within soybean agroecosystems, this trend is especially concerning since laboratory studies have shown that key herbivores exhibit strong thermal adaptive capacity, suggesting a high potential to maintain or increase performance under rising temperatures (Gao et al., 2024). Moreover, analyses of occurrence data indicate that milder winters may facilitate pest persistence and increase the frequency of infestations by relaxing overwintering constraints and promoting selection across seasons (Paul et al., 2024). When combined with the widespread evolution of insecticide resistance in soybean pests (Stacke et al., 2020), these patterns reveal tightly linked climate–ecology–evolution dynamics, underscoring the urgency of anticipating not only greater herbivore pressure but also accelerated pest adaptation in soybean-producing regions.

Soybean (*Glycine max* [L.] Merrill) is a major global source of protein, lipids, and carbohydrates and has become one of the most widely cultivated and consumed legumes worldwide (Spréa et al., 2024). Among its biotic constraints, defoliating caterpillars of the order Lepidoptera rank among the most destructive insect pests, causing severe yield and productivity losses across agricultural systems (Horikoshi et al., 2022). Although the dominant species vary regionally, in the Americas the principal defoliators include *Anticarsia gemmatalis* (Hübner), *Chrysodeixis includens* (Walker), and *Helicoverpa armigera* (Hübner), which are responsible for recurrent outbreaks and substantial economic damage (Souza et al., 2021; Machado et al., 2022).

In Asia, *H. armigera* and *Spodoptera litura* (Fabricius) are among the dominant lepidopteran defoliators of soybean, with extensive evidence of crop damage, migration, and adaptive capacity on soybean hosts (Xu et al., 2022). While in Africa, *Spodoptera frugiperda* (Smith) is now a widespread invasive lepidopteran that feeds on many crops including soybean, with repeated outbreaks, also, *Spodoptera littoralis* (Boisduval) is present in Africa, mediterranean region, and middle east (ElShahed et al., 2023; Togola et al., 2025). Currently, the management of these pests relies mainly on the application of chemical insecticides; however, the prolonged use of such compounds can lead to several detrimental effects on the agroecosystem, including the reduction of natural enemy populations and the outbreak of secondary pest species (Murúa et al., 2018).

As an alternative to mitigate the negative impacts caused by chemical insecticides, several complementary strategies have been adopted, such as the use of *Bt* (*Bacillus thuringiensis*) technologies and other microbial biopesticides, as well as the conservation or release of natural enemies, and the cultivation of resistant plants (Godoy et al., 2022). The incorporation of resistant cultivars stands out as a valuable approach, as it is considered a cost-effective and easily applicable method for farmers (Freitas et al., 2024). Moreover, this strategy can be readily integrated with other control methods within an ecological management framework (Zhou et al., 2024).

In this context, this review discusses the main chemical defense mechanisms associated with soybean genotype resistance to lepidopterans. Furthermore, it presents and classifies soybean varieties resistant to specific species within this insect group. Understanding the complex chemical interactions involved in plant resistance to pests is crucial, as it contributes to the development of new and more tolerant cultivars, thereby reducing the need for insecticides, mitigating negative impacts on the agroecosystem, and slowing the evolution of insect resistance.

To compile the information presented in this review, a comprehensive literature search was carried out to identify studies addressing the defense mechanisms of soybean against lepidopteran pests. The search was conducted in the Web of Science database, covering publications from 2011 to 2025, to include the most recent and relevant research. Various combinations of keywords were used, including *Plant–insect interaction*, *Resistant soybean*, *Plant induced resistance*, *Plant defense*, *Induced defense*, *Resistance*, *Mechanism defense*, *Induced resistance soybean*, *Soybean*, *Chemical defenses of soybeans*, *Spodoptera*, *Defoliating Lepidoptera*, *Soybean and Lepidoptera*, *Soybean Bt*, and *Glycine max and Lepidoptera*, with particular emphasis on the terms *Glycine max*, *Soybean*, and *Lepidoptera*. Search results were organized and screened to retain studies directly addressing plant–insect interactions, induced resistance in soybean, and chemical or physiological defense mechanisms against lepidopterans. Works that focused solely on the chemical characterization of metabolites without relation to defense responses, or those unrelated to soybean resistance to lepidopterans, such as *Bt* toxicology or molecular analyses without emphasis on defense mechanisms, were excluded from the discussion.

## 2 Agronomic context and control of lepidopteran pests in soybean cultivation

### 2.1 Soybean cultivation

Soybean (*Glycine max*) is a leguminous species belonging to the class Magnoliopsida, family Fabaceae, and subfamily Faboideae (Hymowitz and Singh, 1987), which is regarded as a crop of great commercial importance due to the high protein and lipid content of its seeds (Filho et al., 2022). Most of the harvested grains are used for human consumption, primarily in the production of oil, soy milk, flour, and for obtaining soybean meal, an essential ingredient in animal feed (Romero et al., 2020; Maciel et al., 2022). Consequently, soybean is considered the most extensively produced, processed, and traded oilseed crop worldwide (Barboza Martignone et al., 2024).

Due to the high global demand for soybeans, world production in the 2024/25 harvest reached 423.97 million tonnes, cultivated over an area of 146.71 million hectares (United States Department of Agriculture (USDA), 2025c). Currently, Brazil ranks as the world’s largest producer and exporter of soybeans, with an average production of 171.47 million tonnes grown on approximately 47.35 million hectares (Companhia Nacional de Abastecimento (CONAB), 2025). In 2023, following Brazil, the United States (~113 million tonnes), Argentina (~25 million tonnes), China (19 million tonnes), and India (15 million tonnes) were the other major soybean-producing countries (Food and Agriculture Organization (FAO), 2025).

The main consumers of soybeans and soybean meal are concentrated in regions with high levels of livestock production and protein demand, with China standing out as the largest consumer, importing massive quantities to supply its extensive poultry and swine industries, as well as for industrial uses (Zhao et al., 2025). The United States follows, using soybeans both for animal feed and for processing into oil and meal (Gaffield et al., 2024). The European Union, considered collectively, also exhibits high consumption due to intensive livestock systems and indirectly by demand for animal protein (Van Loon et al., 2023).

Although Brazil is one of the world’s leading exporters, it also maintains significant domestic consumption, primarily for feed production (Rezende et al., 2023). In addition, countries such as India, Vietnam, Mexico, and Indonesia rank among major consumers, driven by livestock and population growth with increasing demand for meat, eggs, and milk, trends that highlight the need for new technologies to support sustainable production increase (United States Department of Agriculture (USDA), 2025b).

### 2.2 Major caterpillar pests attacking soybean

Although soybean expansion has been remarkable worldwide, in recent years, the productivity of this legume has been severely affected by various pests, including phytopathogens, nematodes, mites, and especially insects (Hartman et al., 2016; Filho et al., 2022). Insect pests account for the majority of significant yield and economic losses in soybean production (Horikoshi et al., 2022; Ouaarous et al., 2025). The extent of this damage is intensified by several factors, such as tropical climatic conditions and the predominant cropping systems (Chen et al., 2024).

These conditions provide favorable environments and abundant food resources for the development and proliferation of insect pests (Wille et al., 2017). Among the species that attack soybean crops worldwide, those belonging to the orders *Lepidoptera*, *Hemiptera*, and *Coleoptera* are considered the most important, as they feed on different plant parts and tissues, as shown in **Table 1** (de Almeida Barros et al., 2022; Li et al., 2024).

**Table 1:** Main lepidopteran pests that attack soybeans and the plant parts they damage.

Scientific name	Family	Feeding behavior	Global distribution	Impact	Reference
*Helicoverpa armigera*	Noctuidae	Leaves, stems, shoots, flowers, fruits, and pods	Global (Asia, Europe, Africa, Australia; invasive in the Americas)	Very high — causes direct yield losses; epidemic outbreaks reported in South America since 2013	(Kriticos et al., 2015; Coelho et al., 2020)
*Spodoptera frugiperda*	Noctuidae	Leaves, stems, shoots, flowers, fruits, and pods	Global (native to the Americas; invasive in Africa, Asia, and Oceania)	Moderate to high — intense defoliation; soybeans partially compensate but grain filling may be affected at high infestations	(Bernardi et al., 2014; Wyckhuys et al., 2024)
*Spodoptera litura*	Noctuidae	Leaves, stems, flowers, and pods	Asia-Pacific (India, China, Southeast Asia, Japan, Oceania; invasive in parts of Africa)	High — major polyphagous pest of soybean and other crops; causes severe defoliation and yield reduction under favorable conditions	(Cai et al., 2023; Song et al., 2024)
*Spodoptera littoralis*	Noctuidae	Feeds on leaves, flowers, and pods	Africa (widespread in North, East, and parts of West Africa); Mediterranean region; Middle East	High — polyphagous species causing severe defoliation and yield reduction in soybean, cotton, and other legumes; increasing insecticide resistance reported	(ElShahed et al., 2023; El-Refaie et al., 2024; Sebaei, 2024)
*Chrysodeixis includens*	Noctuidae	Leaves	Americas (North, Central, and South), Caribbean; also present in parts of Asia	High — frequent pest causing “windowpane” defoliation and significant economic losses if uncontrolled	(Hodgson et al., 2021)
*Spodoptera eridania*	Noctuidae	Pods and leaves	Americas (mainly South); invasive in Africa.	Moderate — damages leaves and pods but less aggressive than other Spodoptera species	(Sardinha De Souza et al., 2014; Zhang, 2023)
*Helicoverpa zea*	Noctuidae	Leaves, flowers, pods, and seeds	Americas (North, Central, and South)	Moderate to high — attacks reproductive structures; significant in the U.S., but less impact than H. armigera in Brazil and Argentina	(North et al., 2024; Possebom et al., 2025)
*Chloridea virescens*	Noctuidae	Leaves	Americas (mainly affects cotton; secondary occurrence in soybean)	Low — occasionally recorded in soybean but rarely reaches control levels; more relevant in cotton	(Hodgson et al., 2021)
*Elasmopalpus lignosellus*	Pyralidae	Roots	Tropical and subtropical Americas; also reported in other tropical regions	Moderate — can cause stand failures in sandy soils; regionally significant pest	(Marques et al., 2017; Santos et al., 2023)
*Anticarsia gemmatalis*	Noctuidae	Leaves, stems, and pods	Americas (historically the major soybean pest in South America; minor relevance elsewhere)	High — historical defoliator of soybean in Brazil and Argentina	(Hodgson et al., 2021)
*Spodoptera albula*	Noctuidae	Pods	Neotropical region (Central and South America)	Low to moderate — occasional defoliator; more important in beans than soybean	(Murúa et al., 2018)
*Spodoptera cosmioides*	Noctuidae	Pods and leaves	South America (Brazil, Argentina, Paraguay, Uruguay)	Low — rare in soybean; occasional pod damage	(Boiça Júnior et al., 2015a)
*Rachiplusia nu*	Noctuidae	Leaves	South America (mainly Argentina, Uruguay, and southern Brazil)	High — highly aggressive, consumes large amounts of foliage and pods; increasing impact on soybean	(Godói et al., 2025; Godoy et al., 2025)
*Crocidosema aporema*	Tortricidae	Shoots, branches, and pods	South America (Argentina, Uruguay, southern Brazil)	Moderate to high — important pest in the Southern Cone; capable of forming dense populations	(Fernandes et al., 2024)

To date, at least 69 species of Lepidoptera have been recorded from the superfamilies *Pyraloidea*, *Tortricoidea*, *Bombycoidea*, *Hesperioidea*, *Geometroidea*, and *Noctuoidea*, whose larvae have been observed feeding in soybean fields (Formentini et al., 2015). Among these, insect pests belonging to the superfamily *Noctuoidea* have raised particular concern due to their distinctive feeding behavior (Fanela et al., 2020).

Despite the relative diversity of species, the most economically important soybean lepidopterans include the old-world bollworm *H. armigera*, the velvetbean caterpillar *A. gemmatalis* (de Almeida Barros et al., 2022), the tobacco budworm *Chloridea virescens* (Fabricius), and the soybean looper *C. includens* (Wille et al., 2017; Greene et al., 2021; Debnath et al., 2024).

In recent years, however, there has been a notable increase in infestations by *Spodoptera* species, including *S. frugiperda*, *S. eridania* (Stoll), *S. cosmioides* (Walker), and *S. albula* (Walker) (Zhou et al., 2011; Bernardi et al., 2014; Machado et al., 2020). This trend has alarmed farmers due to the high population densities, migratory behavior, and severe feeding damage to leaves, flowers, and pods, resulting in significant economic losses (Specht et al., 2016; Marques et al., 2017; Murúa et al., 2018; Huseth et al., 2021; Justus et al., 2022).

Species of the genus *Spodoptera* represent a significant threat to soybean production across multiple regions worldwide (Machado et al., 2020). Among the main lepidopteran pests, *H. armigera*, a globally distributed species, stands out for attacking the reproductive organs of soybean plants (flowers and pods) as well as vegetative tissues, directly reducing yield and historically causing severe productivity losses even at low population densities (Murúa et al., 2014; Kriticos et al., 2015).

Also widely distributed, *S. frugiperda* exhibits extensive occurrence throughout South America (Brazil, Argentina, Paraguay, and Uruguay), the United States, and more recently China and India, where it causes substantial crop losses due to its high adaptability and rapid population growth (Montezano et al., 2018).

*S. litura*, commonly known as the tobacco cutworm, is a polyphagous noctuid widely distributed across Asia and Oceania, with recent expansion into Africa (Song et al., 2024). It feeds on soybean leaves, stems, flowers, and pods, causing severe defoliation and yield reductions; furthermore its high fecundity, migratory ability, and insecticide resistance make management difficult, ranking it among the most damaging lepidopteran pests of soybean in Asian agroecosystems (Cai et al., 2023).

Likewise, *S. littoralis*, another polyphagous noctuid, is widely distributed across Africa, the Mediterranean basin, and the Middle East (ElShahed et al., 2023). It feeds on soybean leaves, flowers, and pods, causing severe defoliation and significant yield losses (El-Refaie et al., 2024). Owing to its high adaptability and increasing insecticide resistance, *S. littoralis* is considered one of the most important noctuid pests of soybean in Africa, second only to *H. armigera* (Sebaei, 2024).

*C. includens*, regarded as a key soybean pest across the Americas and present in the Caribbean and parts of Asia, is characterized by frequent and persistent defoliation, leading to significant economic losses (Hodgson et al., 2021; Rafael et al., 2021). *S. eridania*, although considered a secondary pest in the United States, displays wide geographic distribution, from South America to Canada, and has emerged as an invasive species in regions of Africa and Asia, causing severe defoliation in crops such as soybean and cassava (Alzate et al., 2025).

*Helicoverpa zea* (Boddie) is notable for feeding on soybean reproductive structures (flowers, pods, and developing seeds), resulting in direct yield losses similar to those caused by *H. armigera*, although this species is more prevalent in North America, where it causes considerable damage, its occurrence in South America is comparatively limited (North et al., 2024; Possebom et al., 2025). *C. virescens*, native to tropical and subtropical regions of the Americas (South, Central, and southern North America), also attack soybeans, though it is historically more important as a key cotton pest, it should not be overlooked in population studies of lepidopteran communities (Hodgson et al., 2021).

*Elasmopalpus lignosellus* (Zeller) is considered a moderate and regionally important pest, particularly in sandy soils of the Brazilian Cerrado and drier southern regions, where it damages soybean stands. In other countries, it causes significant losses in maize, peanut, and bean crops, but soybean plants can often compensate for initial stand loss when infestations occur at low densities (Marques et al., 2017; Santos et al., 2023).

Another species native to tropical and subtropical regions of the Americas, *A. gemmatalis* is highly relevant in Brazil, Argentina, Paraguay, and Uruguay, where it is the primary soybean pest and a voracious defoliator. It also occurs in the southern United States, Mexico, the Caribbean, and Central America, frequently reaching outbreak levels in monoculture systems (Hodgson et al., 2021). *S. cosmioides* (Hill et al., 2025) and *S. albula* (Machado et al., 2020) are primarily found in South America; the former is particularly aggressive, capable of consuming nearly twice the leaf area of soybean as other *Spodoptera* species, while the latter causes more localized and sporadic attacks.

*Rachiplusia nu* (Guenée), restricted to South America, mainly Argentina, Uruguay, Paraguay, and southern Brazil, feeds primarily on foliage, although it may also damage pods. Severe defoliation during reproductive stages can impair grain filling and significantly reduce yields, especially during outbreak events (Godói et al., 2025; Godoy et al., 2025). *Crocidosema aporema* (Walsingham) is another regional pest typical of the Southern Cone, usually considered secondary, it can become highly damaging during localized outbreaks, especially in late-season plantings; furthermore, sporadic infestations have led to significant losses in pods and fertile branches in southern Brazil and Argentina (Fernandes et al., 2024).

Collectively, these species highlight the global impact of Lepidoptera on soybean production, emphasizing the urgent need for Integrated Pest Management strategies to mitigate yield losses and promote sustainable crop production worldwide.

### 2.3 Management of caterpillars in soybean

For many years, the use of synthetic insecticides has been the primary strategy adopted worldwide for managing lepidopteran pests in soybean and other leguminous crops (Marques et al., 2016; Huseth et al., 2021). The main advantages driving their intensive application include a broad spectrum of activity, allowing control of multiple pest species, low initial cost, ease of use, and high effectiveness (Plata-Rueda et al., 2020).

The most widely used insecticides belong to the neonicotinoid, organophosphate, and pyrethroid classes, which act as sodium channel blockers and neurotoxins, causing hyperexcitation of the nervous system, paralysis, and ultimately insect death (Pereira et al., 2023). However, excessive chemical spraying has led to serious environmental and agronomic issues, such as the selection of resistant insect populations, secondary pest outbreaks, and negative impacts on natural enemies and biological control agents (Ren et al., 2023).

In this context, the introduction of genetically modified plants has become a widely adopted approach to reduce insecticide use and its negative consequences, while significantly contributing to advances in Integrated Pest Management programs (Barcellos et al., 2023; Carrière and Tabashnik, 2023). Among these, transgenic *Bt* soybean cultivars stand out. These plants express *cry* genes from *Bacillus thuringiensis* (Berliner), which encode Cry1Ac toxins that reduce larval feeding, disrupt development, and increase mortality rates of lepidopteran pests (Silva et al., 2014).

Currently, *Bt* soybean is the primary control method for key lepidopteran pests damaging soybean in South America, while adoption in other major producing countries such as the United States, China, and India remains limited (Machado et al., 2020). This technology provides strong protection against defoliators such as *A. gemmatalis*, *C. includens*, *C. virescens*, and *H. armigera* (Bacalhau et al., 2020).

The contrasting adoption patterns between regions highlight substantial differences in pest management strategies. In Brazil, *Bt* soybean cultivation reached approximately 94% of the total soybean area during the 2023/2024 growing season, reflecting the widespread reliance on this technology for lepidopteran control (Bueno et al., 2025). By comparison, soybean biotechnology adoption in the United States is dominated by herbicide-resistant traits, accounting for about 96% of soybean acreage in 2025. Notably, U.S. agricultural statistics do not report a specific insect-resistant or *Bt* soybean category, which is consistent with the limited or near-negligible commercial adoption of *Bt* soybean in that country (United States Department of Agriculture (USDA), 2025a).

Nevertheless, *Bt* soybean exhibits limited toxicity against *Spodoptera* species, including *S. frugiperda*, *S. eridania*, *S. albula*, and *S. cosmioides*, due to poor compatibility between *Bt* proteins and midgut receptors, high detoxification capacity, and natural tolerance *(*Bernardi et al., 2014*).* Therefore, complementary strategies such as *Bt* protein pyramiding, technology stacking, and Integrated Pest Management are required to maintain control efficacy (Horikoshi et al., 2021; Machado et al., 2022).

At the molecular level, the insecticidal activity of Cry toxins relies on their high insertion of toxins into the lipid bilayer and the formation of transmembrane pores, which are specific interactions with receptors located in the larval midgut epithelium (Jurat-Fuentes et al., 2021). Following solubilization and proteolytic activation in the alkaline gut lumen, Cry toxins initially bind to low-affinity receptors in the brush border membrane, including cadherins, aminopeptidase N (APN), and alkaline phosphatases (ALP). This initial interaction induces conformational changes and promotes toxin oligomerization at the apical membrane surface (Núñez-Ramírez et al., 2020).

The resulting oligomers subsequently interact with high-affinity receptors, particularly ATP-binding cassette (ABC) transporters such as ABCC2 and ABCA2, which actively mediate toxin insertion into the lipid bilayer and transmembrane pore formation (Amezian et al., 2024). Pore formation disrupts membrane integrity, leading to osmotic imbalance, midgut epithelial cell lysis, and larval death (Jin et al., 2026). Recent studies indicate that changes in the expression or functionality of ABC transporters significantly reduce Cry toxin insertion efficiency, contributing to *Bt* tolerance or resistance in lepidopteran species, including *Spodoptera* spp (Liu et al., 2024a, 2025). In addition, mitogen-activated protein kinase (MAPK) signaling has been implicated in the regulation of midgut genes associated with *Bt* responses, conferring physiological plasticity and facilitating adaptation to Cry toxins (Guo et al., 2020).

Although *Bt* cultivars have played a central role in controlling primary soybean pests, widespread and repeated use of this technology can exert strong selection pressure, accelerating the evolution of resistance in target insects (Ongaratto et al., 2021). To mitigate this risk, refuge areas with non-*Bt* soybean are recommended to delay resistance development and preserve long-term effectiveness (Reisig, 2017).

**Figure 1:**
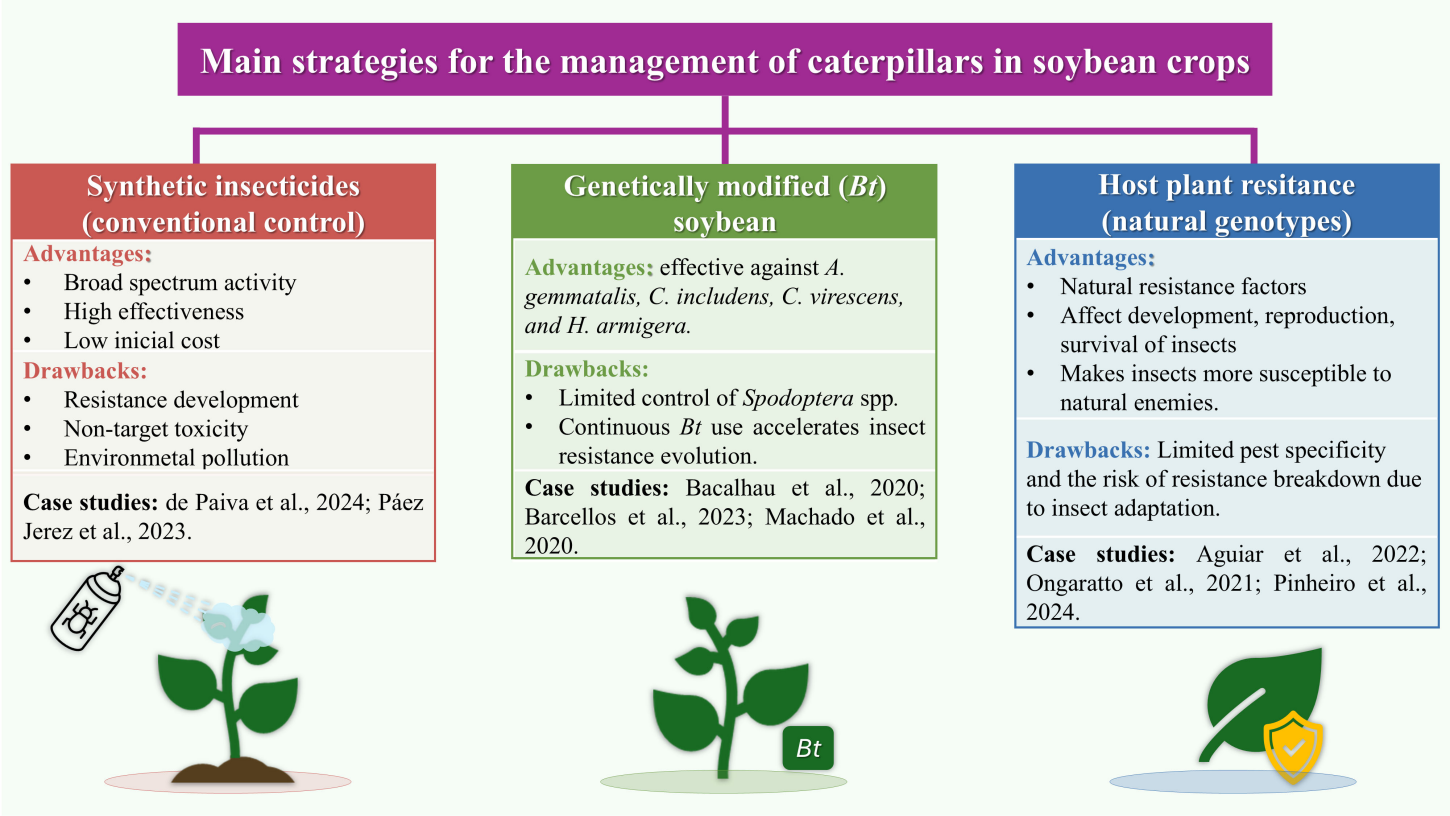
Main strategies for the management of lepidopteran caterpillars in soybean crops. The figure summarizes conventional chemical control, genetically modified (*Bt*) soybean, and host plant resistance based on natural plant genotypes. For each strategy, major advantages and limitations are indicated, together with representative case studies reported in the literature. Synthetic insecticides provide broad-spectrum control but are associated with resistance development and environmental concerns (Páez Jerez et al., 2023; de Paiva et al., 2024) *Bt* soybean effectively controls key pests such as *A. gemmatalis* and *C. includens*, although its efficacy may decline due to resistance evolution in *Spodoptera* spp (Bacalhau et al., 2020; Machado et al., 2020; Barcellos et al., 2023). Host plant resistance relies on constitutive or inducible traits that negatively affect insect behavior and performance, but its effectiveness may vary among genotypes and environmental conditions (Ongaratto et al., 2021; Aguiar et al., 2022; Pinheiro et al., 2024).

Another promising alternative is the use of soybean genotypes that naturally express higher levels of insect resistance (Ulhoa et al., 2023). These resistance factors vary according to insect species and plant genotype. Resistant plants possess complex and sophisticated defense mechanisms that negatively affect multiple biological parameters of insects, including development, reproduction, and survival (Canassa et al., 2017; Morando et al., 2017). Consequently, these genotypes can reduce pest populations without major ecological imbalances, leaving insects more vulnerable to natural enemies (de Freitas et al., 2018). Thus, host plant resistance represents a valuable and environmentally friendly strategy that complements other Integrated Pest Management tools, enhancing the sustainability and efficiency of pest management systems. The main strategies currently used for caterpillar management including conventional chemical control, *Bt* transgenic cultivars, and naturally resistant genotypes, are summarized in **Figure 1**, along with their respective advantages and limitations.

## 3 Mechanisms and strategies of soybean defense against lepidopteran caterpillars

### 3.1 Plant resistance mechanisms

Throughout their long evolutionary history with herbivorous insects, plants have developed a wide range of defense mechanisms to protect themselves from attack (Kant et al., 2015). These strategies are generally classified as constitutive or induced. Constitutive defenses are always present, regardless of herbivory, whereas induced defenses are activated only after insect attack (Chen et al., 2018). Studies indicate that some constitutive defenses can be further strengthened during herbivory (Traw and Dawson, 2002; Bixenmann et al., 2016). However, induced defenses are particularly important for host-plant resistance because they incur lower metabolic costs and exhibit greater specificity against herbivores (Gordy et al., 2015; Acevedo et al., 2021).

Two major types of inducible defenses have been described in plants: direct and indirect defenses (Gols, 2014). Direct defenses involve traits that act directly on the herbivore, including structural barriers (e.g., epicuticular features and pubescence/trichomes), cell-wall reinforcement (thickening and lignification), mineral-based defenses, and toxic/antinutritional chemistry such as secondary metabolites, protease inhibitors, and enzymes that impair insect growth and development (Chen, 2008; War et al., 2012; Gols, 2014; Wari et al., 2022).

As an illustrative example in soybean, resistance to the Neotropical brown stink bug *Euschistus heros* has been reported in cultivars characterized by reduced host attractiveness and significantly lower feeding activity on pods and seeds relative to susceptible cultivars (Lucini et al., 2021).

Similarly, several soybean genotypes exhibit resistance to the velvetbean caterpillar *Anticarsia gemmatalis*, manifested as decreased feeding and consumption rates, along with reduced larval growth and overall performance. These insect responses support the incorporation of such resistance-related traits into soybean breeding programs (Piubelli et al., 2005). In other examples, at the molecular level, silencing GmVQ58 enhanced resistance to *S. litura* through a WRKY-associated defense module (Li et al., 2020), and gene-editing/omics validation of GmCDPK38 further showed that manipulating a central signaling regulator can strengthen soybean resistance to common cutworm (Li et al., 2022).

Indirect defenses, in contrast, reduce herbivory by recruiting natural enemies through the emission of herbivore-induced plant volatiles (HIPVs) (War et al., 2012; Gols, 2014; Aljbory and Chen, 2018). In soybean at field-level, methyl salicylate has been used to attract natural enemies in soybean systems (e.g., in aphid-associated contexts) (Mallinger et al., 2011), and more recently, application of methyl salicylate and (E,E)-α-farnesene increased the abundance of stink bug egg parasitoids (Scelionidae) and enhanced egg parasitisation in soybean fields (Michereff et al., 2024).

**Figure 2:**
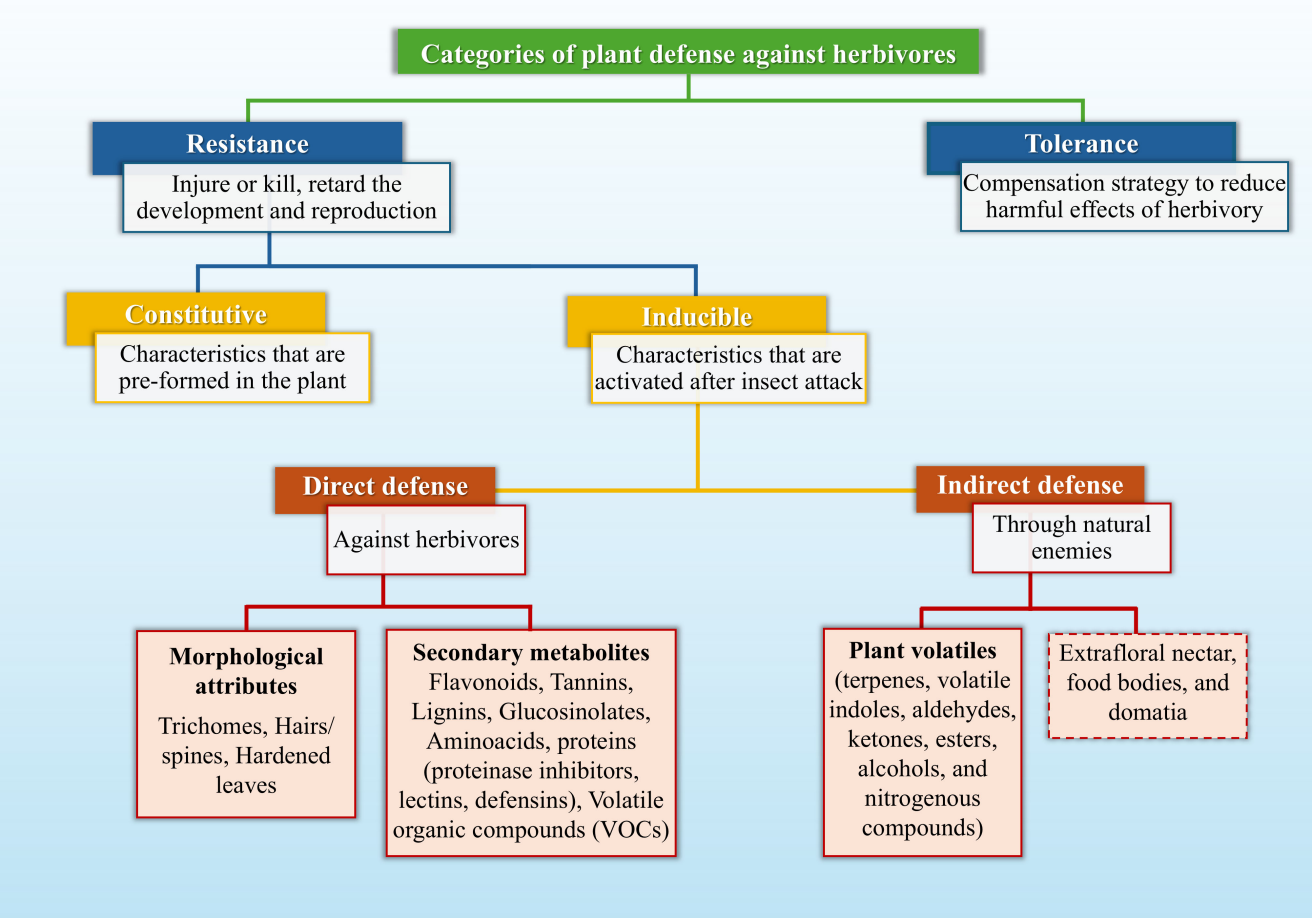
Conceptual framework illustrating the categories of plant defense against herbivores. Plant defenses are classified as constitutive or inducible, the latter being activated in response to herbivore attack and particularly relevant for host-plant resistance. Inducible defenses comprise two main subcategories: direct defenses, which directly affect herbivore feeding, growth, development, and survival through structural and chemical traits, and indirect defenses, which reduce herbivory by attracting natural enemies via herbivore-induced volatile compounds. Extrafloral nectar, food bodies, and domatia are examples of constitutive indirect defenses (highlighted in the dashed box). The framework also distinguishes resistance mechanisms, which limit damage, from tolerance traits that minimize yield loss per unit of damage.

**Figure 2** summarizes a conceptual framework for studying plant resistance. This model distinguishes between resistance, defined as plant traits that restrict damage, and tolerance, characterized by traits that minimize yield loss per unit of damage. Resistance is further divided into constitutive or inducible, and into direct or indirect mechanisms. Such a framework provides a clear representation of how plants mitigate herbivore impacts and helps integrate fundamental and applied perspectives in the study of plant–insect interactions, thereby guiding research toward elucidating the underlying mechanisms of plant resistance.

### 3.2 Resistant soybean varieties

Exploring natural plant resistance is a key strategy to reduce the impact of herbivores on crop yield and quality. This resistance arises from the expression of various physicochemical traits that hinder the efficient use of plants as hosts by insect pests (Stout, 2014; Das et al., 2022). The traditional approach to developing and applying resistant varieties within Integrated Pest Management involves screening germplasm, classifying resistance mechanisms, introducing resistance genes into agronomically suitable backgrounds, and integrating these varieties into management programs (Stout, 2014; Das et al., 2022; López et al., 2022).

In classifying plant resistance to herbivores, experimental techniques generally focus on three major categories: antibiosis, antixenosis, and tolerance (Smith, 2005). Antibiosis occurs when insect biology is adversely affected, impairing growth, development, reproduction, and survival. Common biological indicators of this resistance type are reduced body size and weight, prolonged life cycle, adult deformities, and decreased fecundity (Hondelmann et al., 2020).

Antixenosis relates to behavioral deterrence, plants become less attractive for feeding or oviposition, these evaluations include leaf consumption (e.g., eaten area or weight), feeding preference, and oviposition choice (Stec et al., 2021). Whereas tolerance refers to the plant’s ability to withstand or recover from pest damage without affecting insect biology or behavior (Queiroz et al., 2020; Ongaratto et al., 2021).

From an experimental perspective, antibiosis is primarily distinguished through insect growth and development indices (e.g., body weight, developmental duration, survival, and fecundity), whereas antixenosis is assessed using behavioral preference assays, such as feeding deterrence, leaf consumption, and oviposition choice (Bansal et al., 2021; Ongaratto et al., 2021; Kaler et al., 2025).

Recent studies have evaluated soybean cultivars for lepidopteran resistance (**Table 2**). For instance, the varieties PI 548402, BAUS 102, DSB34, MACS 1493, and RSC 11–03 expressed antixenosis resistance to *S. litura* (Yano et al., 2021; Mathpal et al., 2022), while PI 171451, PI 274453, IAC 18, IAC 23, L1-1-01, BMX Ícone IPRO, and Tec Irga 6070 RR displayed antixenosis against *C. includens* (Schlick-Souza et al., 2017; Gobbi et al., 2024).

**Table 2:** Soybean cultivars evaluated for resistance to lepidopteran pests.

Genotype	Resistent to:	Reference
Benso 1RR and BMX Turbo RR	*C. includens*	(Wille et al., 2017)
BRS 7270 IPRO	*S. cosmioides*	(Queiroz et al., 2020)
D75-10169	*A. gemmatalis*	(Ongaratto et al., 2021)
IAC 17	*A. gemmatalis*	(Gómez et al., 2018, 2020; Ongaratto et al., 2021)
	*H. armigera*	(Coelho et al., 2020)
IAC 23	*C. includens*	(Schlick-Souza et al., 2017)
IAC 24 and IAC 74-2832	*A. gemmatalis*	(Ongaratto et al., 2021)
IAC 100	*C. virescens*	(Almeida et al., 2017; Boiça Júnior et al., 2022)
	*A. gemmatalis*	(Boiça Júnior et al., 2015a; Ongaratto et al., 2021)
	*S. cosmioides*	(Boiça Júnior et al., 2015a; de Queiroz et al., 2020; Queiroz et al., 2020)
	*S. frugiperda*	(Boiça Júnior et al., 2015a, 2017)
IAC 78-2318	*A. gemmatalis*	(Ongaratto et al., 2021)
IAC 19	*A. gemmatalis*	(Ongaratto et al., 2021)
	*H. armigera*	(Coelho et al., 2020)
L1-1-01	*C. includens*	(Schlick-Souza et al., 2017)
Lamar	*S. litura*	(Li et al., 2016, 2017; Zhang et al., 2018)
PI 171451	*H. armigera*	(Coelho et al., 2020)
PI 226782	*S. cosmioides*	(Queiroz et al., 2020)
PI 227682	*S. cosmioides*	(Aguiar et al., 2022; Boiça Júnior et al., 2015b; de Freitas et al., 2018; de Queiroz et al., 2020)
	*S. eridania*	(Souza et al., 2014)
PI 227687	*S. cosmioides*	(Boiça Júnior et al., 2015a; de Queiroz et al., 2020; Queiroz et al., 2020)
	*H. armigera*	(Coelho et al., 2020)
	*S. eridania*	(Souza et al., 2014)
	*S. frugiperda*	(Boiça Júnior et al., 2017; Souza et al., 2021)
	*A. gemmatalis*	(Souza et al., 2021)
PI 229358 and PI 274453	*H. armigera*	(Coelho et al., 2020)
PI 274454	*A. gemmatalis*	(Ongaratto et al., 2021)
	*H*. *armigera*	(Coelho et al., 2020)
PI 548402 (Peking)	*S. litura*	(Yano et al., 2021)
‘TMG 1179’ RR, ‘TMG 133’ RR, and ‘TMG 7062’ IPRO	*A. gemmatalis*	(Ongaratto et al., 2021)
UFUS Carajás, UFUS Impacta, UFUS Milionária, and UFUS Xavante	*S. cosmioides*	(Aguiar et al., 2022)
BAUS 102, DSB34, MACS 1493, and RSC 11-03	*S. litura*	(Mathpal et al., 2022)
BMX Icone Ipro and Tec Irga 6070 RR	*C. includens*	(Gobbi et al., 2024)
BRS 8383 IPRO, BRS 1074 IPRO, BRS 1061 IPRO, BRS 7180 IPRO, BRS 9383 IPRO, BRS 8980 IPRO, BRS 1003 IPRO, BRS 523, and BRS 543 RR	*A. gemmatalis*	(Lima et al., 2024)
G17 and G19	*S. litura*	(Krisnawati et al., 2025)

Other authors reported that IAC 100, PI 227682, and PI 227687 exhibited antixenosis to *S. cosmioides*, associated with trichome density and leaf coloration (de Queiroz et al., 2020). Antibiosis was also identified in PI 227687, PI 226782, IAC 100, and BRS 7270 IPRO against the same species. Similarly, PI 227687 and IAC 100 showed antibiosis to *S. frugiperda* (Boiça Júnior et al., 2017), and PI 227687, PI 227682, IAC 100, and DM 339 demonstrated antibiosis against *S. eridania* (Souza et al., 2014).

Although antixenosis and antibiosis are traditionally considered independent categories, they often overlap in practice, making them difficult to separate conceptually (Stout, 2013). Most resistant plants possess traits that are simultaneously repellent, deterrent, toxic, or antinutritional. Consequently, many secondary metabolites exert both antixenotic and antibiotic effects (Stenberg and Muola, 2017). For example, the genotypes IAC 74-2832, PI 274454, BRS 8383 IPRO, BRS 1074 IPRO, BRS 1061 IPRO, BRS 7180 IPRO, BRS 9383 IPRO, BRS 8980 IPRO, and BRS 1003 IPRO exhibited antixenosis or antibiosis against *A. gemmatalis*, while IAC 24, BRS 523, BRS 543 RR, G17, and G19 expressed both mechanisms (Lima et al., 2024; Krisnawati et al., 2025). Likewise, PI 227687, PI 274454, and IAC 19 were identified as resistant to *H. armigera* due to reduced leaf consumption and adverse effects on insect biology (Coelho et al., 2020).

Overall, studies summarized in **Table 2** indicate that PI 227687, PI 227682, and IAC 100 are the most promising genetic sources for developing high-yielding and pest-resistant soybean cultivars, however, numerous biotic and abiotic factors can influence the expression of resistance (Stout, 2013; Boiça Júnior et al., 2022). Therefore, field validation under diverse environmental conditions remains essential to confirm laboratory findings (Boiça Júnior et al., 2015b).

In conclusion, natural plant resistance is a strategic resource for Integrated Pest Management, particularly in soybean cultivation. Although classical categories such as antibiosis and antixenosis remain useful for screening and classifying resistant genotypes, their isolated application is often insufficient to capture the complexity of the underlying biochemical, genetic, and ecological mechanisms.

### 3.3 Key metabolites involved in soybean defense against lepidopterans

**Figure 3:**
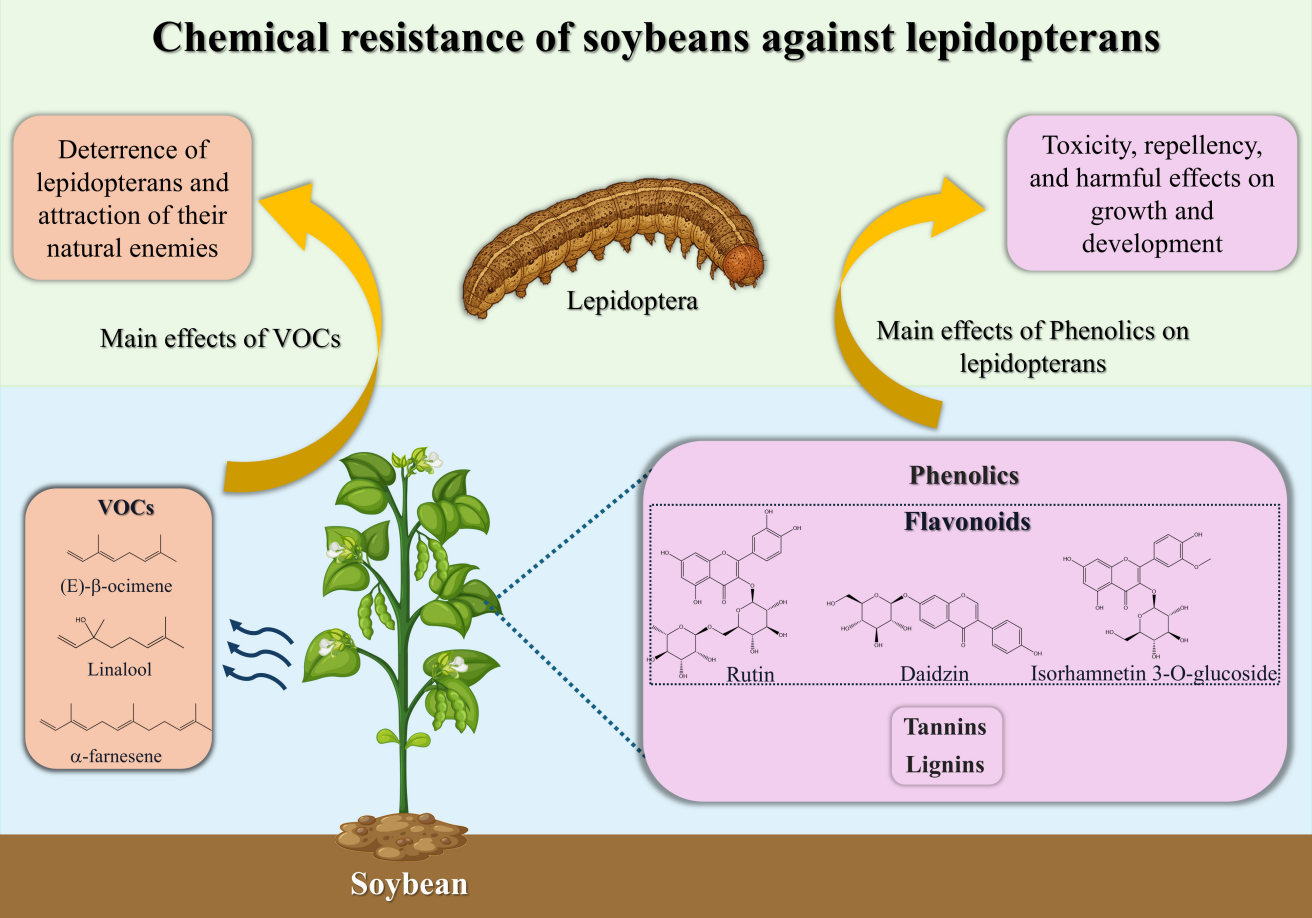
Schematic summary of major secondary metabolites involved in soybean defense against lepidopteran pests. The figure highlights constitutive and inducible chemical defenses, including phenolics, flavonoids, isoflavones, and VOCs, and their principal effects on lepidopterans.

The previous section summarized soybean genotypes exhibiting resistance to lepidopterans through antibiosis and antixenosis. However, defensive strategies vary greatly among species and depend on multiple factors, including genotype, plant tissue, herbivore type, and tritrophic interactions within the plant–insect–environment system (Fürstenberg-Hägg et al., 2013; Stenberg and Muola, 2017). This section discusses the main defensive chemical compounds identified in soybean genotypes over the last decade, emphasizing secondary metabolites such as phenolic compounds and volatile organic compounds (VOCs). The main defensive compounds identified in soybean and their effects on lepidopteran pests are summarized in **Figure 3**.

Secondary metabolites are organic molecules derived from primary metabolism that play critical roles in plant defense and signaling (Jha and Mohamed, 2022). They participate in mechanisms of resistance, tolerance to biotic and abiotic stress, and ecological interactions with pollinators, microorganisms, and herbivores (Ku et al., 2020). Their abundance and composition vary among species and tissues, reflecting metabolic processes that suppress insect growth and development (Divekar et al., 2022). Mechanistically, these compounds impair herbivore performance by inhibiting enzymes, disrupting nutrient assimilation, generating oxidative stress, or interfering with detoxification pathways, thereby linking metabolite profiles to biological resistance outcomes (Divekar et al., 2025).

Soybean tissues contain diverse secondary metabolites, among which flavonoids are the most abundant class (da Graça et al., 2016; Silva et al., 2018). Found in multiple plant organs at variable concentrations, flavonoids regulate oxidative stress, mediate hormonal signaling, protect against UV radiation, and act as defensive agents against pathogens and insects (Romani et al., 2003; Perlatti et al., 2015; da Graça et al., 2016; Silva et al., 2018). Flavonoids such as quercetin, kaempferol, and genistein can reduce larval growth by inhibiting midgut proteases, increasing oxidative imbalance, and limiting detoxification efficiency, which directly translates into antibiosis against several lepidopteran species (Gautam et al., 2023; Pereira et al., 2024).

The constitutive resistance of certain soybean varieties to *S. cosmioides* is associated with the content of specific lipids and isoflavones in leaves (Aguiar et al., 2022). Comparative analyses between resistant (IAC 17) and susceptible (UFV105) genotypes identified compounds such as methylquercetins, quercetin glycosides, genistein, kaempferol, and daidzein, especially kaempferol and quercetin derivatives, as key chemical markers of resistance to *A. gemmatalis* (Gómez et al., 2018). These flavonoids act predominantly through antibiosis, since quercetin and kaempferol negatively affect protein digestion and nutrient assimilation, while isoflavones such as genistein and daidzein exhibit direct toxicity and can interfere with hormonal pathways related to insect growth and metamorphosis. These physiological effects provide a functional basis for the reduction in larval performance observed in resistant genotypes (Pereira et al., 2024).

Studies using resistant and susceptible soybean lines have revealed genes and proteins involved in the response to larval feeding and the biosynthesis of secondary metabolites (Shim et al., 2024). Compounds such as rutin and isorhamnetin glycosides are constitutively produced in resistant genotypes (IAC 17, IAC 100), and herbivory by *A. gemmatalis* induces the expression of flavonol-synthesis-related genes, including *FLS* and *O-methyltransferases*, suggesting potential targets for breeding programs (Pinheiro et al., 2024). Similarly, proteins differentially expressed in response to *S. litura* feeding are linked to secondary metabolite biosynthesis, redox homeostasis, and cellular signaling, highlighting them as candidates for genetic manipulation to enhance soybean resistance (Yadav and Singh, 2024).

The analysis of phenolic, tannin, fiber, and lignin content in soybean leaves, pods, and seeds demonstrated that high concentrations of these compounds in the cultivar IAC 100 contribute to resistance against *C. virescens*, with tannins identified as one of the main chemical defense mechanisms (Boiça Júnior et al., 2022). Tannins exert multiple defensive effects, including protein precipitation in the insect gut, reduced digestibility of plant tissues, and impaired nitrogen assimilation, all of which reduce larval efficiency in converting food into biomass (Barbehenn and Peter Constabel, 2011; Zheng et al., 2022; Gupta et al., 2025). Resistance was also influenced by leaf age and plant developmental stage: higher levels of rutin, isoquercitrin, daidzin, daidzein, and hesperidin were found in mature leaves of PI 227687 during the reproductive phase, making them less suitable for *A. gemmatalis* and *S. frugiperda* development (Souza et al., 2021).

Induced defenses reduce herbivore performance and feeding preference by affecting their physiology and behavior (Chen et al., 2018). For soybean, induced resistance to defoliators has been widely documented (Yousefi-Taemeh et al., 2021). It was observed that both mechanical injury and insect feeding increased phenolic production in soybean leaves; however, only larvae that fed on insect-damaged leaves showed impaired development, indicating a herbivore-specific signaling response (Peruca et al., 2018). The induction of phenolic compounds has been shown to prevent feeding and oviposition, and to increase the activity of detoxifying enzymes in the midgut of insects, indicating their toxic nature (Usha Rani and Pratyusha, 2014; Movva and Pathipati, 2017).

Environmental factors such as UV-B radiation can also modulate soybean leaf chemistry, thus, in plants exposed to natural sunlight, herbivory by *A. gemmatalis* was correlated with ethylene emission and induction of isoflavonoids, as well as alterations in quercetin and kaempferol glycosides (Dillon et al., 2017, 2018). Similarly, exogenous elicitors derived from insect oral secretions can trigger plant defenses: application of *S. litura* gut extracts induced daidzein, genistein, formononetin, and their conjugates (Nakata et al., 2016). The addition of exogenous elicitors in soybean plants can trigger resistance against specific lepidopteran pests (e.g., common soybean defoliators) and reduce their fitness, allowing the regulation of pest populations by natural enemies without compromising seed production (Chen et al., 2018).

Silicon (Si) supplementation has also been shown to enhance plant resistance, since both herbivory and Si treatment increased phenolic content in soybean (Acevedo et al., 2021). Likewise, Si accumulation in soybean leaves reduced the growth rate of *Heliothis punctigera* (Wallengren), suggesting that Si acts as an inducible defense against herbivores (Johnson et al., 2020).

Volatile organic compounds represent another major class of defensive metabolites, mediating indirect defenses by attracting natural enemies, facilitating plant–plant communication, and enhancing tolerance to stress (Vivaldo et al., 2017). Soybean VOCs include terpenes, esters, aldehydes, and alcohols (Kim et al., 2020; Song et al., 2022). These compounds can repel herbivores, alter feeding patterns, reduce oviposition (Makhlouf et al., 2024; Mobarak et al., 2025), and attract parasitoids (Park et al., 2025), thereby integrating chemical and ecological layers of defense. Yet, some insects can suppress or evade VOCs-based defenses, for instance, *H. zea* larvae secrete salivary enzymes that induce stomatal closure, reducing VOCs emission from soybean leaves (Lin et al., 2021).

Herbivore-induced changes in VOCs profiles differ among soybean genotypes, e.g., *A. gemmatalis* feeding increased the emission of (*Z*)-3-hexen-1-ol, 1-octen-3-ol, (*Z*)-β-ocimene, β-linalool, MeSA, indole, and jasmonates (Rocha et al., 2023). In another study, *S. litura* feeding induced terpenes such as linalool, α-farnesene, and (*E*)-β-ocimene, the latter synthesized by *GmOCS*, which plays a key role in insect repellence and represents a promising gene for soybean improvement (Han et al., 2023).

Soybean defenses were further shown to be enhanced when *Spodoptera exigua* (Hübner) larvae infected with nucleopolyhedrovirus (NPV) released three volatile organic compounds, 3-octanone, (*E*)-2-hexenal, and 2-butyl-1,1,3-trimethyl-cyclohexane, that attract parasitoids (Wan et al., 2017). Moreover, subsequent research demonstrated that *C. includens* herbivory in two soybean lines promoted different VOCs emissions between isogenic and transgenic plants. Compounds such as (*Z*)-3-hexenyl-2-methylbutyrate, (*Z*)-jasmonate, β-linalool, and TMTT were induced, and female *Meteorus rubens* (Nees von Esenbeck) preferred odors from herbivore-damaged transgenic plants. These results indicate that genetic modification can influence tritrophic interactions among plants, pests, and natural enemies (Strapasson et al., 2016).

In summary, soybean resistance to lepidopterans cannot be fully explained by the classical categories of antibiosis and antixenosis alone. The diversity of secondary metabolites, such as flavonoids, phenolics, and VOCs, reveals a sophisticated chemical arsenal acting both constitutively and inducibly to modulate herbivore behavior, physiology, and ecological interactions. The variability of metabolite expression across genotypes, tissues, and phenological stages underscores the need for integrated approaches that consider ecological, genetic, and physiological factors. Advancing the characterization of bioactive compounds and their regulatory genes represents a promising path toward breeding more resilient cultivars capable of maintaining productivity while reducing chemical input dependence, thus aligning with sustainable agriculture goals.

### 3.4 Genome editing and gene-based engineering for enhanced soybean defense

Beyond conventional breeding, genome editing has emerged as an additional strategy for improving soybean insect resistance. Early platforms such as zinc-finger nucleases (ZFNs) and transcription activator-like effector nucleases (TALENs) were introduced in 2011 and 2014; however, CRISPR/Cas has become the preferred approach due to its simplicity, efficiency, and versatility (Freitas-Alves et al., 2025). Importantly, genome editing can support sustainable soybean improvement by accelerating the development of lines with enhanced tolerance to abiotic stress and improved resistance to biotic constraints, including pests and diseases, thereby contributing to reduced pesticide inputs (Kim et al., 2025).

Several gene-based strategies illustrate this potential. For example, overexpression of soybean trypsin inhibitors (e.g., KTI7 and BBI5) increased trypsin and chymotrypsin inhibitory activities and reduced *H. zea* larval weight (Sultana et al., 2023). Similarly, transgenic soybean expressing the codon-optimized cry1c* gene from *Bacillus thuringiensis* impaired larval growth, decreased body weight, and reduced survival of multiple chewing pests, including *Leguminivora glycinivorella*, *Spodoptera exigua*, *S. litura*, and *Mythimna separata* (Fang et al., 2024). More recently, the transgenic soybean event CAL-16, expressing a Cry1Ab–Vip3A fusion protein, achieved complete mortality of neonates of *H. armigera*, *S. litura*, *Agrotis ipsilon*, *S. exigua*, and *S. frugiperda*, and provided full field protection against *L. glycinivorella* (Pan et al., 2025).

Notably, recent evidence also indicates that soybean insect resistance can be strengthened not only through insecticidal or antinutritional proteins, but also via regulatory control of specialized metabolism. For instance, mutation of a soybean UDP-glycosyltransferase (GmUGT) altered flavonoid biosynthesis and enhanced resistance to chewing lepidopteran pests (Zhang et al., 2022). In parallel, overexpression of GmOCS ((*E*)-β-ocimene synthase) increased terpene volatile emission and reduced feeding by (and/or repelled) *S. litura*, illustrating a metabolite-centered engineering route via volatile terpenoids (Han et al., 2023).

Consistently, metabolomics of soybean lines carrying insect-resistance Quantitative Trait Loci showed that resistance is associated with broad metabolic reprogramming, with flavonoids/isoflavonoids highlighted as key chemical classes linked to these loci ( (Yousefi-Taemeh et al., 2021). Another promising and still underexplored chemical layer in soybean–lepidopteran interactions is the phenolamide (hydroxycinnamic acid amide, HCAA) pathway, which has been increasingly recognized as a rapidly inducible defense module in plants, being tightly connected to jasmonate signaling and polyamine metabolism (Roumani et al., 2021, 2022).

Collectively, these studies reinforce that soybean insect resistance can be pursued through complementary routes like protein-based defenses, genetic engineering, and targeted regulation of defensive metabolic pathways (e.g., flavonoids, terpenoid volatiles, and phenolamides) to support durable and sustainable pest management The future of developing resistant cultivars will rely on integrating empirical knowledge, technological innovation, and field validation, ensuring greater efficiency, sustainability, and adaptability of agricultural systems to emerging phytosanitary challenges.

## 4 Perspectives

The evidence gathered in this review highlights that soybean’s chemical resistance to lepidopterans represents a promising tool to reduce yield losses and decrease dependence on synthetic insecticides. Future research should prioritize the in-depth characterization of secondary metabolites, particularly flavonoids, phenolics, and volatile compounds, associated with soybean resistance (Gómez et al., 2018; Makhlouf et al., 2024). This effort should be supported by omics technologies (genomics, transcriptomics, metabolomics, and lipidomics) and bioinformatic analyses capable of identifying molecular targets and resistance biomarkers (Aguiar et al., 2022).

At the same time, there is a need to expand field experiments that account for biotic, abiotic, and multitrophic interactions to validate laboratory findings and better understand the ecological role of chemical defenses in agroecosystems (Dillon et al., 2017; Souza et al., 2021). The application of exogenous elicitors and resistance inducers, such as jasmonates and silicon, combined with the exploration of volatile compounds for attracting natural enemies, represents a practical avenue within Integrated Pest Management strategies (Strapasson et al., 2016; Acevedo et al., 2021).

Additionally, investigating key insect detoxification enzymes and mechanisms that enable herbivores to neutralize or metabolize soybean defensive compounds is essential (Fonseca et al., 2023). Understanding these adaptive pathways will support the development of strategies to counteract herbivore metabolic plasticity, thus maintaining the long-term effectiveness of plant defenses (Bhattacharyya et al., 2007).

Future perspectives point to a multidisciplinary approach in which the integration of plant breeding, biotechnology, chemical ecology, and agroecological practices will foster the development of more resilient soybean genotypes. Striking an optimal balance between productivity and chemical defenses, supported by advances in biotechnological tools, will be essential to address emerging pest pressures and ensure the long-term sustainability of soybean cultivation.

## 5 Final considerations

Plant defense mechanisms vary widely among plant–insect systems and depend on the type of herbivory. Defenses that are effective against one species may be secondary or ineffective against another. In the soybean–lepidopteran system reviewed here, cultivars such as IAC 100 and PI 227687 have shown resistance to key pests including *A. gemmatalis*, *S. cosmioides*, and *S. frugiperda*. Secondary metabolites, particularly phenolic compounds like flavonoids and VOCs appear to play central roles in these interactions. However, to validate these findings, field experiments are required, taking into account abiotic and biotic factors, as well as multitrophic interactions that influence plant defensive responses.

Sustainable pest management is best achieved through the integration of resistant plant varieties within an Integrated Pest Management framework, combined with good agricultural practices, pest monitoring, biological control, agroecological management, and the judicious use of environmentally safer pesticides. A comprehensive understanding of the global factors influencing soybean–lepidopteran interactions, together with advances in genomics, proteomics, metabolomics, lipidomics, and bioinformatics, will enable the development of new high-yielding and pest-resistant soybean genotypes.
